# Combined atrial fibrillation ablation and left atrial appendage closure: Watchman vs. LAmbre devices

**DOI:** 10.3389/fcvm.2022.1011037

**Published:** 2022-11-02

**Authors:** Jin-Yan Ke, Lu-Shen Jin, Yuan-Nan Lin, Jing Xu, Wei-Ke Liu, Jia-Yang Fu, Ling Li, Yi-Lian Chen, Yi-Xuan Qiu, Yue-Chun Li

**Affiliations:** Department of Cardiology, Second Affiliated Hospital and Yuying Children's Hospital of Wenzhou Medical University, Wenzhou, China

**Keywords:** left atrial appendage closure, radiofrequency catheter ablation, atrial fibrillation, Watchman, LAmbre

## Abstract

**Background:**

Left atrial appendage closure (LAAC) combined with radiofrequency catheter ablation is an emerging one-stop hybrid procedure for non-valvular atrial fibrillation (AF). This study was performed to compare the efficacy and safety of the Watchman device vs. the LAmbre device for this combined procedure.

**Methods:**

Two hundred and thirty two patients with AF who underwent the combined procedure were enrolled and divided into two subgroups depending on the device choice: the Watchman-combined group (*n* = 118) and the LAmbre-combined group (*n* = 114). The periprocedural and follow-up adverse events in both groups were documented.

**Results:**

The mean CHA_2_DS_2_-VASc score and HAS-BLED score in the Watchman-combined group and LAmbre-combined group were 3.7 ± 1.5 vs. 3.8 ± 1.5 and 2.5 ± 1.1 vs. 2.3 ± 1.1, respectively (all *P* > 0.05). Successful LAAC was achieved in all patients. The rate of major periprocedural complications and AF recurrence at 6 months post-procedure were similar between the Watchman-combined group and LAmbre-combined group (0.8 vs. 0.9%, *P* = 1.00; 22.0 vs. 15.8%, *P* = 0.23). During 2.6 ±0 .7 vs.1.6 ± 1.6 years follow-up, the rate of major clinical adverse events, including stroke and major bleeding, were comparable between the Watchman-combined group and the LAmbre-combined group (2.6 vs. 1.1% per 100 patient-years, *P* = 0.33). The intraprocedural peri-device leakage (PDL) rate was similar between the Watchman-combined group and the LAmbre-combined group (5.1 vs. 6.1%, *P* = 0.73), but the PDL rate was significantly higher at 3–6 months transesophageal echocardiography (TEE) follow-up than the intraprocedural PDL rate in both groups (21.6 vs. 5.1%; 36.6 vs. 6.1%, respectively), with a more obvious increase in minimal PDL rate in the LAmbre-combined group than the Watchman-combined group (36.6 vs. 21.6%, *P* < 0.05).

**Conclusion:**

The Watchman and LAmbre devices were comparable in efficacy and safety for the combined procedure. The minimal PDL rate at short-term TEE follow-up was higher in the LAmbre-combined group than the Watchman-combined group.

## Introduction

Radiofrequency catheter ablation (RFCA) combined with left atrial appendage (LAA) closure (LAAC) is an emerging one-stop procedure. This combined procedure not only improves patients' symptoms but also prevents stroke and reduces the risk of bleeding in patients with non-valvular atrial fibrillation (AF) compared with oral anticoagulants (OAC) (1–3). There are two main types of LAAC devices currently available for clinical application: “plug” and “disk” devices. Several large clinical trials have demonstrated that LAAC with implantation of the Watchman device (Boston Scientific, Marlborough, MA, USA) effectively reduced the incidence of all-cause stroke, bleeding events, and adverse cardiovascular and cerebrovascular events (4–7). The LAmbre device (Lifetech Scientific, Shenzhen, China) is a type of “disk” device with a classic double-disk structure. Previous studies have confirmed that LAAC with the LAmbre is as effective and safe as the “plug” device (8–10). However, no comparative studies have focused on the outcomes of the one-stop combined procedure using the Watchman and LAmbre devices. The purpose of this study was to compare the clinical effectiveness and safety of the one-stop combined procedure performed with the Watchman and LAmbre devices.

## Study methods

### Study population

A total of 232 consecutive patients with non-valvular AF who underwent LAAC combined with RFCA at the Second Affiliated Hospital of Wenzhou Medical University from August 2018 to October 2021 were included in this retrospective study. Depending on the device choice, the patients were divided into two subgroups: the Watchman-combined group and the LAmbre-combined group. The inclusion criteria were symptomatic non-valvular AF, age ≥18 years, with the CHA2DS2-VASc score of ≥2 and satisfaction of any of the following criteria: (1) history of bleeding, (2) difficulty maintaining a stable international normalized ratio with warfarin, (3) poor compliance with OAC therapy, or (4) the HAS-BLED score of ≥3. The exclusion criteria are as follows: (1) a history of artificial heart valve replacement, or (2) a thrombus found in the LAA.

In this study, all patients' baseline characteristics, periprocedural, and follow-up data were collected. The study conformed to the Declaration of Helsinki and was approved by the Ethics Committee of the Second Affiliated Hospital of Wenzhou Medical University. Informed consent was obtained from each patient for this study.

### Device introduction

#### Watchman device

The nickel-titanium alloy skeleton of the occlusion device is shaped like a “jellyfish” and covered with a polyester fiber membrane with barbs to hold it in the LAA. There are five sizes of the device (21, 24, 27, 30, and 33 mm) to accommodate different races and sizes of LAA. In clinical practice, the Watchman device that is 10% to 20% larger than the LAA ostial width is usually chosen to achieve complete occlusion and less postoperative residual shunting.

#### LAmbre device

The LAmbre consists of a disk and an umbrella-shaped structure with barbs and a waist attached in the middle. The disk surface is 4 to 6 mm larger than the umbrella-shaped structure, and the device has different models ranging from 16 to 36 mm. The device is usually 2 to 6 mm larger than the LAA. The delivery sheath tube is sent to the proximal end of the LAA. The LAmbre device has two advantages: (1) The small delivery sheath tube facilitates operation and also reduces the risk of intraoperative thrombosis, perforation to pericardial tamponade, and puncture injury. (2) The device has a small disk and a large cover, which makes it suitable for the treatment of patients with multiple lobes and other complex LAA anatomy (8).

#### Preoperative preparation

All patients underwent preoperative uninterrupted OAC treatment and transesophageal echocardiography (TEE) to rule out LAA thrombosis.

#### Left atrial appendage closure

The details of the LAAC procedure have been previously reported (9, 11, 12). Briefly, the procedure was performed after the patient had received local or general anesthesia. To obtain an activated clotting time of 250–300 s, patients received intravenous heparin during the combined procedure (13). The morphology and size of the LAA were delineated by preoperative TEE and LAA angiogram to guide the selection of the LAAC device. The device was selected at the discretion of the operators. The appropriately sized device was pushed to the LAA through the delivery sheath. Then a tug test was conducted under fluoroscopy to check the stability of the device. Successful LAAC was defined as proper deployment and implantation of the LAA occlusion device and PDL ~3 mm by intraprocedural angiography/TEE, whereas complete occlusion is defined as no PDL.

### Radiofrequency catheter ablation

Ablation was performed with radiofrequency energy. All patients underwent standard pulmonary vein isolation (PVI). Individualized additional linear ablations (left anterior wall line, mitral isthmus line, left roof line, superior vena cava isolation, tricuspid isthmus line, or other linear lesions as considered appropriate) were performed based on the demands for persistent AF, longstanding persistent AF, or redo ablation procedures. RFCA was performed either before or after LAAC according to the operator's experience. Nevertheless, for patients who underwent LAAC before RFCA, RFCA was performed carefully to avoid adverse events such as displacement or embolization of the device when it was close to the newly implanted device.

### Post-procedural anticoagulation and follow-up

All patients routinely received anticoagulation therapy for at least 3 months after the combined procedure. The anticoagulation regimen was usually either warfarin or novel OACs depending on the individual patient. Follow-up was performed during conventional clinical visits, and a 12-lead electrocardiogram was obtained at 1, 3, and 6 months and every 6 months thereafter. Additionally, 24-h Holter monitoring was performed at 3 and 6 months and every 6 months thereafter. AF recurrence was defined as AF rhythm detected by electrocardiography or 24-h Holter monitoring in the absence of antiarrhythmic drugs after a 3-month blank period. TEE was performed 3–6 months after the combined procedure to assess device stability, and LAA sealing as well as to rule out pericardial effusion (PE) and device-related thrombus (DRT). If the TEE examination suggested satisfying LAA sealing (PDL <3 mm or no PDL at any angle) and no DRT during follow-up, the patient was subsequently treated with single antiplatelet therapy (SAT) for the long-term. If DRT was detected during follow-up, anticoagulation was restarted and TEE was performed every 3 months until the thrombus disappeared. The decision to maintain or discontinue anticoagulation in patients who did not have follow-up imaging was made on an individual basis depending on LAAC procedure records at the discretion of the physician.

### Events definition

The major periprocedural complications include: hemorrhagic or ischemic stroke and death related to the procedure, procedural thrombosis formation, device-related complications requiring open surgery or major endovascular intervention, any bleeding related to the combined procedure need for transfusions of red blood cell (RBC) ≥2 units within 24 h and PE requiring pericardiocentesis. The minor periprocedural adverse events include mild PE (<10 mm) (14), and puncture complications without intervention. During follow-up, major clinical adverse events include all-cause death, hemorrhagic or ischemic stroke, transient ischemic attack (TIA), systemic embolism, and major bleeding requiring surgery or transfusion of RBC. Adverse events were assessed according to the Munich consensus document on definitions, endpoints, and data collection requirements (15), the 2017 Cardiovascular and Stroke Endpoint Definitions (16), and the Bleeding Academic Research Consortium (BARC) (17).

### Statistical analysis

Statistical analyses were performed by R software version 3.6.1 (R Foundation for Statistical Computing, Vienna, Austria). Continuous variables are presented as mean ± standard deviation, and categorical variables are presented as count and percentage. The chi-square test or Fisher's exact test was used to compare categorical variables. Continuous variables were compared between two groups by the *t*-test or Mann–Whitney U test. The incidence rates of adverse events are reported as annualized rates (events/patient-years of follow-up). The Kaplan–Meier method was used for the graphical analysis of time-dependent events. The log-rank (Mantel-Cox) test was used to compare event curves. A *P* value of <0.05 was considered statistically significant.

## Results

### Study population

In total, 232 patients who underwent LAAC combined with RFCA procedure using the Watchman (Watchman-combined group, *n* = 118) or LAmbre (LAmbre-combined group, *n* = 114) device were enrolled in this retrospective study. The patients' baseline characteristics are shown in Table 1. All relevant baseline characteristics of the Watchman-combined group and LAmbre-combined group were comparable, particularly the risk of stroke and bleeding (mean CHA_2_DS_2_-VASc score of 3.7 ± 1.5 vs. 3.8 ± 1.5, *P* = 0.56; mean HAS-BLED score of 2.5 ± 1.1 vs. 2.3 ± 1.1, *P* = 0.29). A history of catheter ablation was present in 15 (12.7%) patients in the Watchman-combined group and 12 (10.5%) patients in the LAmbre-combined group (*P* = 0.60) (Table 1).

**Table 1:** Baseline characteristics.

**Variable**	**Watchman-combined (*n* = 118)**	**LAmbre-combined (*n* = 114)**	***p*-values**
**Demographics and clinical features**
Ages (years)	67.4 ± 8.8	68.4 ± 8.4	0.38
Body mass index (kg/m^2^)	25.0 ± 3.3	24.6 ± 3.3	0.30
Female gender, *n* (%)	49 (41.5%)	46 (40.4%)	0.86
LVEF (%)	62.6 ± 7.9	61.5 ± 10.0	0.39
Coronary artery disease, *n* (%)	17 (14.4%)	20 (17.5%)	0.51
Prior PCI/CAGB, *n* (%)	5 (4.2%)	9 (7.9%)	0.24
Prior ablation, *n* (%)	15 (12.7%)	12 (10.5%)	0.60
**AF phenotypes**
Paroxysmal AF, *n* (%)	52 (44.1%)	42 (36.8%)	0.26
Persistent AF, *n* (%)	38 (32.2%)	41 (36.0%)	0.55
Long-standing persistent AF, *n* (%)	28 (23.7%)	31 (27.2%)	0.54
**CHA2DS2-VASc score**	3.7 ± 1.5	3.8 ± 1.5	0.56
Prior stroke/TIA, *n* (%)	35 (29.7%)	26 (22.8%)	0.24
Congestive heart failure, *n* (%)	27 (22.9%)	27 (23.7%)	0.89
Arterial hypertension, *n* (%)	90 (76.3%)	75 (65.8%)	0.08
Age >75 (years), *n* (%)	29 (24.6%)	25 (21.9%)	0.63
Vascular disease, *n* (%)	48 (40.7%)	44 (38.6%)	0.75
Diabetes mellitus, *n* (%)	36 (30.5%)	28 (24.6%)	0.31
**HAS-BELD score**	2.5 ± 1.1	2.3 ± 1.1	0.29
Prior bleeding, *n* (%)	10 (8.5%)	13 (11.4%)	0.46
Labile INR, *n* (%)	33 (28.0%)	26 (22.8%)	0.37
Kidney disease, *n* (%)	12 (10.2%)	7 (6.1%)	0.26
Liver disease, *n* (%)	7 (5.9%)	5 (4.4%)	0.60
Drugs with predisposition to bleeding, *n* (%)	22 (18.6%)	26 (22.8%)	0.43
Alcohol abuse, *n* (%)	16 (13.6%)	22 (19.3%)	0.24

LVEF, left ventricular ejection fraction; PCI, percutaneous coronary intervention; CABG, coronary artery bypass grafting; AF, atrial fibrillation; TIA, transient ischemic attack; INR, international normalized ratio.

### Procedural characteristics

PVI was performed in 50 (42.4%) and 39 (34.2%) patients in the Watchman-combined group and LAmbre-combined group, respectively, while the remaining 68 (57.6%) and 75 (65.8%) patients underwent PVI plus linear ablation. There was no significant difference in the procedural duration between the Watchman-combined group and the LAmbre-combined group (160.8 ± 51.9 min vs. 170.5 ± 48.6 min, *P* = 0.06). As presented in Table 2, LAA angiography showed that a cauliflower LAA morphology was the most common morphological type in the two groups. In addition, LAA ostial width and depth were greater in the LAmbre-combined group than the Watchman-combined group (LAA ostial width 25.9 ± 4.3 mm vs. 23.0 ± 3.1 mm; LAA depth 26.2 ± 5.7 mm vs. 22.4 ± 4.0 mm, all *P* < 0.05). Two patients in each group crossed over LAAC device types to the other group due to complex LAA anatomy. The device implantation success rate was 100% in both groups. More patients in the LAmbre-combined group needed to change device size due to suboptimal device implantation than the Watchman-combined group [15/114 (13.2%) vs. 6/118 (5.1%), *P* < 0.05]. The intraprocedural PDL rate was similar between the Watchman-combined group and the LAmbre-combined group (5.1 vs. 6.1%, *P* = 0.73), neither group detected PDL of >3 mm. In terms of cost-effectiveness between the two groups, the Watchman-combined group had higher procedure costs than the LAmbre-combined group ($ 16083.6 ± 1434.4 vs. $ 15253.9 ± 1174.2, *P* < 0.05).

**Table 2:** Procedural.

**Variable**	**Watchman-combined (*n* = 118)**	**LAmbre-combined (*n* = 114)**	***p*-values**
**Anesthesia**
General anesthesia, *n* (%)	7 (5.9%)	4 (3.5%)	0.39
Local anesthesia, *n* (%)	111 (94.1%)	110 (96.5%)	0.39
**Procedure time (min)**	160.8 ± 51.9	170.5 ± 48.6	0.06
**Procedure costs ($)**	16083.6 ± 1434.4	15253.9 ± 1174.2	<0.05
**LAAC characteristics**
Device size (mm)	27.7 ± 3.1	32.9 ± 4.0	<0.05
LAA ostial width (mm)	23.0 ± 3.1	25.9 ± 4.3	<0.05
LAA depth (mm)	22.4 ± 4.0	26.2 ± 5.7	<0.05
**LAA morphology**
Windsock, *n* (%)	16 (13.6%)	18 (15.8%)	0.63
Cauliflower, *n* (%)	82 (69.5%)	62 (54.4%)	0.02
Chicken, *n* (%)	15 (12.7%)	28 (24.6%)	0.02
Cactus, *n* (%)	5 (4.2%)	2 (1.8%)	0.45
Implant success, *n* (%)	118 (100%)	114 (100%)	1.00
Complete occlusion, *n* (%)	112 (94.9%)	107 (93.9%)	0.73
PDL, *n* (%)	6 (5.1%)	7 (6.1%)	0.73
≥3 mm, *n* (%)	0	0	-
<3 mm, *n* (%)	6 (5.1%)	7 (6.1%)	0.73
Device released for one time, *n* (%)	112 (94.9%)	99 (86.8%)	<0.05
**RFCA characteristics**
RFCA prior to LAAC of AF, *n* (%)	102 (86.4%)	111 (97.4%)	<0.05
RFCA, *n* (%)	118 (100%)	114 (100%)	1.00
PVI only, n (%)	50 (42.4%)	39 (34.2%)	0.20
PVI plus linear ablation, *n* (%)	68 (57.6%)	75 (65.8%)	0.20

LAAC, left atrial appendage closure; LAA, left atrial appendage; AF, atrial fibrillation; PDL, peri-device leakage; RFCA, radiofrequency catheter ablation; PVI, pulmonary vein isolation.

### Periprocedural events

The periprocedural complications are shown in Table 3. No patients in either group developed procedure-related death, stroke, device dislodgment, or procedural thrombosis formation. One patient in each group developed severe PE as detected by transthoracic echocardiography within 24 h after the combined procedure. The effusion improved after aggressive pericardial puncture, drainage and blood transfusion. Mild PE (<10 mm) was detected by transthoracic echocardiography before discharge in 15 (12.7%) patients in the Watchman-combined group and 18 (15.8%) patients in the LAmbre-combined group (*P* = 0.50). The rate of minor puncture complications was numerically higher in the Watchman-combined group than in the LAmbre-combined group, but there was no statistical difference (6.8 vs. 3.5%, *P* = 0.26).

**Table 3:** Peri-procedural adverse events and antithrombotic therapy.

**Variable**	**Watchman-combined (*n* = 118)**	**LAmbre-combined (*n* = 114)**	***p*-values**
**Peri-procedural serious adverse events**, ***n*** **(%)**	1 (0.8%)	1 (0.9%)	1.00
Procedure-related death, *n* (%)	0	0	-
Procedure-related stroke, *n* (%)	0	0	-
Procedural thrombosis formation, *n* (%)	0	0	-
Major bleeding, *n* (%)	1 (0.8%)	1 (0.9%)	1.00
PE requiring pericardiocentesis	1 (0.8%)	1 (0.9%)	1.00
Device dislodgment, *n* (%)	0	0	-
**Anti-thrombotic therapy post-procedure**
Warfarin, *n* (%)	14 (11.9%)	15 (13.2%)	0.77
Rivaroxaban, *n* (%)	103 (87.3%)	98 (86.0%)	0.63
Dabigatran, *n* (%)	1 (0.8%)	1 (0.9%)	1.00

PE, pericardial effusion.

### Follow-up

#### Antithrombotic therapy management and TEE

The anticoagulation therapy after the combined procedure is summarized in Table 3. Excluding patients who refused or were not suitable for TEE examination, data on TEE imaging within 3–6 months postoperatively were available in 74.6% (88/118) of patients in the Watchman-combined group and 81.6% (93/114) of patients in the LAmbre-combined group (*P* = 0.20, Table 4). The rate of complete LAA sealing was higher in the Watchman-combined group than in the LAmbre-combined group [(78.4% (69/88) vs. 63.4% (59/93); *P* < 0.05)]. The rate of minimal PDL during TEE follow-up was significantly higher in the LAmbre-combined group than in the Watchman-combined group (36.6 vs. 21.6%, *P* < 0.05) (Table 4). In addition, the rate of mild PE <10 mm was similar between the Watchman-combined group and the LAmbre-combined group (4.2 vs. 5.3%; *P* = 0.71). One patient in the Watchman-combined group developed a DRT event, and the thrombus dissolved completely after changing the anticoagulant regimen to warfarin. Based on the TEE examination, 98.9% (87/88) of patients in the Watchman-combined group and 100% (93/93) in the LAmbre-combined group were treated with SAT. At 6-month postoperatively, 93.2% (110/118) of patients in the Watchman-combined group and 95.6% (109/114) of patients in the LAmbre-combined group discontinued their anticoagulants, which was based on the physician's judgment for patients who did not undergo TEE examination at follow-up.

**Table 4:** Follow-up.

**Variable**	**Watchman-combined (*****n*** = **118)**		**LAmbre-combined (*****n*** = **114)**		***P*-value**
Follow-up in y, mean ± SD	2.6 ± 0.7		1.6 ± 1.6		<0.05
AF recurrence 6-month post-procedure, *n* (%)	26 (22.0%)		18 (15.8%)		0.23
**TEE follow-up (3-6month)**
TEE performed, *n* (%)	88 (74.6%)		93 (81.6%)		0.20
DRT, *n* (%)	1 (1.1%)		0		1.00
Complete LAA sealing	69 (78.4%)		59 (63.4%)		<0.05
PDL, > 3 mm	0		0		-
PDL, ≤3 mm	19 (21.6%)		34 (36.6%)		<0.05
Device dislodgment, *n* (%)	0		0		-
PE, *n* (%)	5 (4.2%)		6 (5.3%)		0.71
**Adverse events in follow-up**	Events/patient-y	Observed rate	Events/patient-y	Observed rate	
Serious adverse events	8/303	2.6 (1.1–5.1)	2/182	1.1 (0.1–3.9)	0.33
All-cause death	1/303	0.3 (0.01–1.8)	0/182	0 (0.0–2.0)	1.00
Cardiovascular/unexplained death	0/303	0 (0.0–1.2)	0/182	0 (0.0–2.0)	-
Stroke and TIA (any)	3/303	1.0 (0.2–2.9)	0/182	0 (0.0–2.0)	0.30
Systemic embolism	0/303	0 (0.0–1.2)	0/182	0 (0.0–2.0)	-
Any bleeding	18/303	5.9 (3.6–9.2)	16/182	8.8 (5.1–13.9)	0.87
Major bleeding	4/303	1.3 (0.4–3.3)	2/182	1.1 (0.1–3.9)	1.00

TEE, transesophageal echocardiography; DRT, device-related thrombus; LAA, left atrial appendage; PDL, peri-device leak; PE, pericardial effusion; TIA, transient ischemic attack.

### AF recurrence

The recurrence rate of AF was similar between both groups at the 6-month follow-up (Table 4). The AF recurrence rate was 34.7% in the Watchman-combined group during 2.6 ± 0.7 years and 26.3% in the LAmbre-combined group during 1.6 ± 1.6 years.

No patients underwent repeat ablation except for two patients in the Watchman-combined group and three patients in the LAmbre-combined group.

### Clinical adverse events

**Figure 1:**
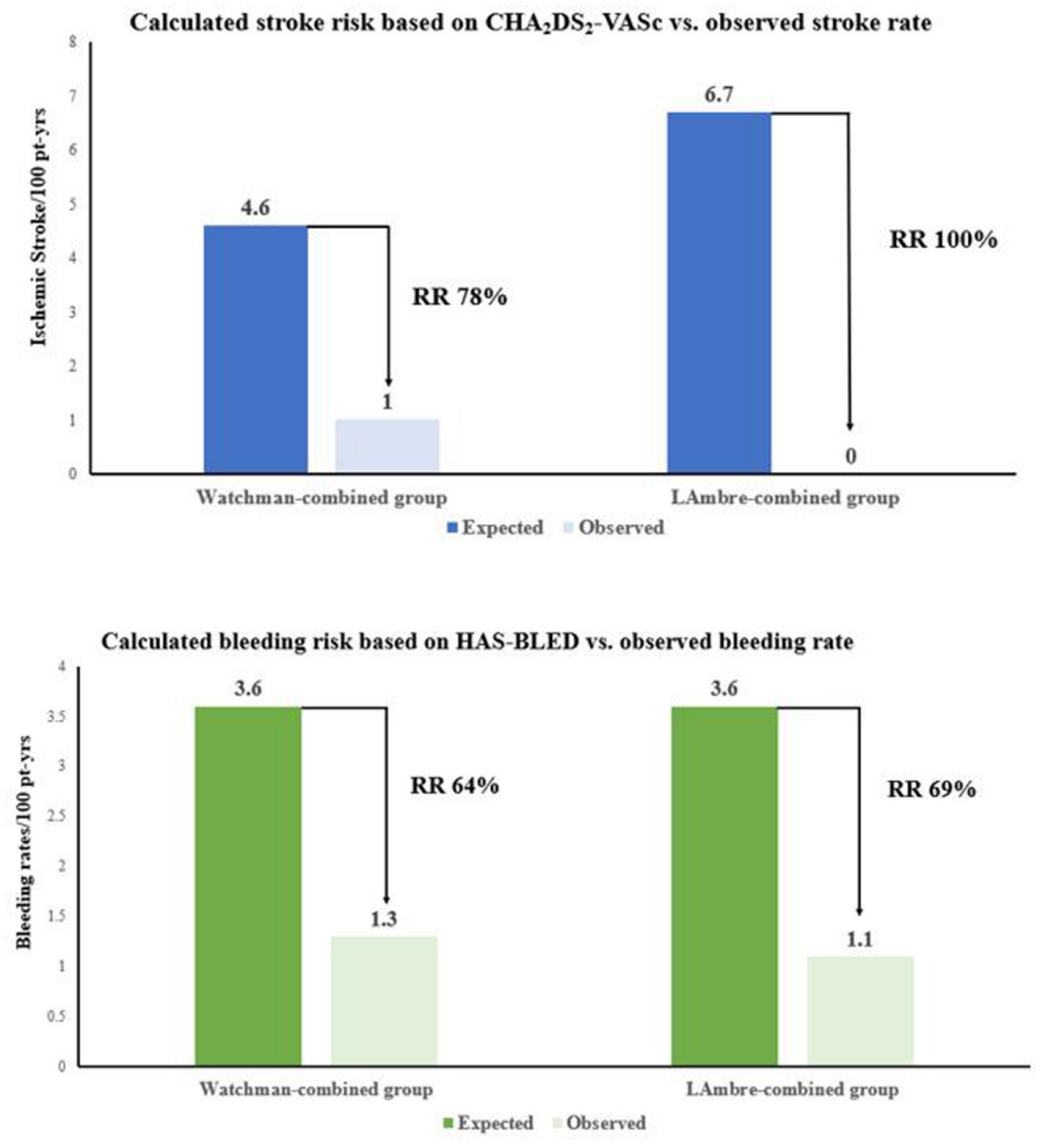
Effectiveness of combined procedure with Watchman and LAmbre devices in reducing stroke and bleeding rate.

**Figure 2:**
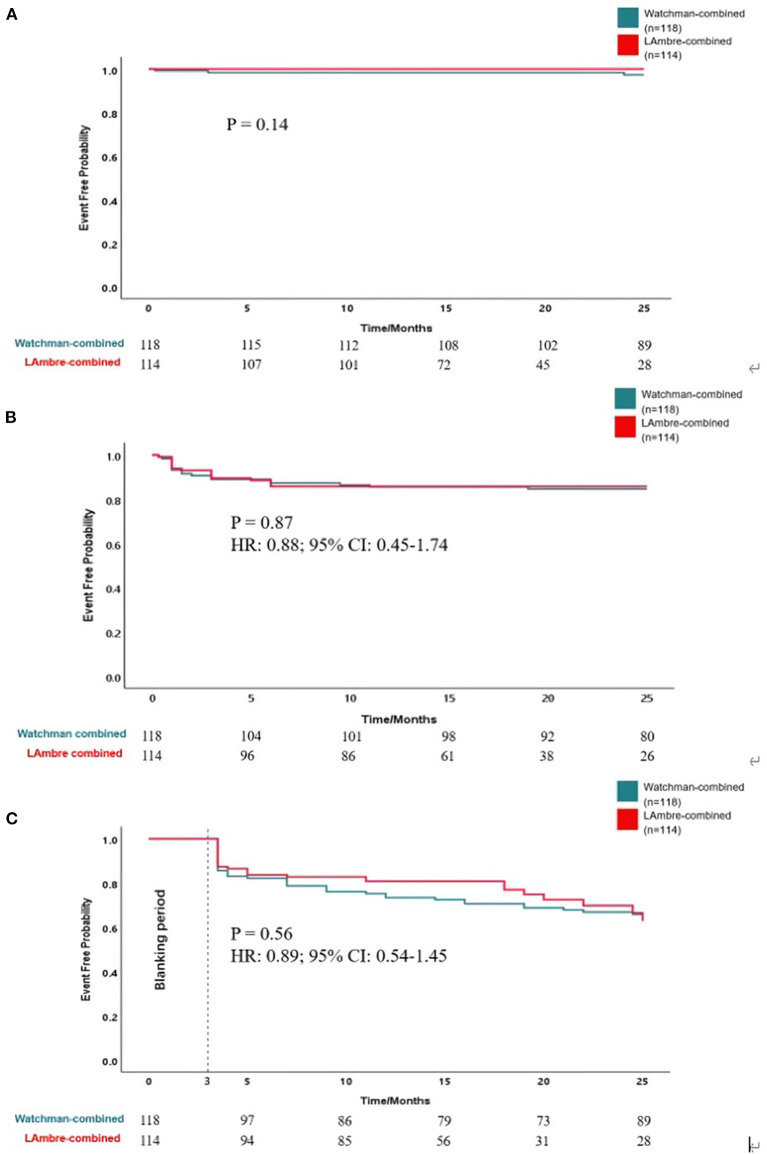
Kaplan-Meier curves of **(A)** freedom from all-cause stroke, TIA and death, **(B)** freedom from bleeding events, and **(C)** freedom from atrial fibrillation recurrence.

The clinical adverse events are summarized in Table 4. Rates of the adverse events were calculated as the number of events per 100 patient-years of follow-up. Meier curves of the adverse events are shown in **Figure 2**. All-cause death, all-cause stroke/TIA, and systemic embolism occurred in 4 of 118 patients with the Watchman-combined group during 303 patient-years, 1.3% per 100 patient-years, and no such adverse events occurred in the LAmbre-combined group during 182 patient-years. Any bleeding events occurred 18/303 (5.9%) in the Watchman-combined group and 16/182 (8.8%) in the LAmbre-combined group (HR, 0.88; 95% CI, 0.45–1.74; *P* = 0.87). Compared with estimated annual rates from CHA_2_DS_2_-VASc (18) score and HAS-BLED (19) score, the decline of stroke and bleeding events rates in the two combined groups was documented in Figure 1.

## Discussion

To the best of our knowledge, this study is the first to compare the combined procedural success and mid-term clinical outcome between the Watchman and LAmbre devices. The main findings of this study were that the efficacy and safety of Watchman and LAmbre devices were comparable in the combined procedure. The rate of PDL was significantly higher at 3–6 months TEE follow-up than in the periprocedural period, and it was higher in the LAmbre-combined group than the Watchman-combined group.

RFCA has become an effective method for rhythm control in patients with NVAF (20), but there is a risk of AF recurrence after RFCA (21). Current guidelines recommend continued anticoagulation for patients with NVAF who have a high CHA_2_DS_2_-VASc score even if ablation is successfully performed (20). LAAC has been proven to have efficacy in preventing strokes equal to OACs for patients with NVAF (20, 22). Because of the similarities between RFCA and LAAC in terms of atrial septal puncture, anesthesia, and postoperative anticoagulation, the combination therapy of AF ablation with LAAC not only avoids the added risk of multiple procedures but also controls AF symptoms and permanently reduces the stroke risk. Clinically available LAAC devices are divided into two types: “plug” and “disk” devices. Of the “plug” devices, the Watchman is the most commonly used in clinical practice, and several studies have confirmed its safety and efficacy in hybrid procedures (1–3, 12). The LAmbre has been proposed to be feasible in the LAAC procedure, (9, 23, 24) but data regarding its use in the combined procedure are limited. At present, there is still controversy regarding whether the Watchman or LAmbre device is superior or inferior in the combined procedure.

The device implantation success rate was 100% for both devices in the combined procedure, similar to other studies of the Watchman [91 (25), 95 (26), and 98.3% (27)] and LAmbre [99.3 (9) and 100% (26)] devices. The LAmbre may be more suitable than the Watchman in patients with complex anatomies, such as an LAA with a larger size, and this was reflected in our study. The LAA ostial diameter (25.9 ± 4.3 mm vs. 23.0 ± 3.1 mm, *P* < 0.05) and depth (26.2 ± 5.7 mm vs. 22.4 ± 4.0 mm, *P* < 0.05) were larger in the LAmbre-combined group than in the Watchman-combined group, which is attributed to the LAmbre device's variety of available sizes and unique stabilization mechanism by catching the LAA trabeculations with its eight claws (24). However, the rate of one-time device release in the LAmbre-combined group was significantly lower than that in the Watchman-combined group, mainly for the following reasons: (1) the LAmbre outer disk impacted the nearby tissues, such as the mitral valve and pulmonary vein; (2) due to the greater variety in the LAmbre device, it was more difficult to choose the most optimal device size; and (3) more patients had a large LAA ostial diameter and complex LAA morphology in the LAmbre-combined group, which affected the selection of the device.

The rate of major periprocedural complications was similar in both groups and was lower than in other combined procedure studies [2.1% (3) and 8.6% (28)]. The adverse event included one case of pericardial tamponade within 24 h after the combined procedure in both groups which was not caused by the device but was related to the procedure.

In both groups, the rate of PDL was significantly higher at TEE follow-up than in the periprocedural period. Additionally, the rate of PDL was significantly higher in the LAmbre-combined group than Watchman-combined group [36.6% (34/93) vs. 21.6% (19/88), *P* < 0.05], however, no PDL >3 mm was detected in either group. LAA measuring for device selection was conducted without CCTA, which is the gold standard. This could be a part of the explanation for the high rate of PDL. Another reason for the high rate of PDL during the follow-up period in the LAmbre-combined group may be related to more patients with complex LAA anatomies and thus LAAC procedure is more difficult (more co-axiality requirements). Acute edematous change of the left atrial ridge (LAR) caused by ablation may have been one of the causes. The EWOLUTION/WASP data showed a similar increase in the incidence of PDL during follow-up, but the increase was greater in the combined procedure than in Watchman LAAC alone (29). Ren et al. (30) reported that swelling of the LAR was observed in patients who underwent the combined procedure, and the swelling was characterized by a huge change in the diameter of the outer ostium but only a slight change in the inner ostium. Therefore, these lesions may affect the measurement of the LAmbre device but have little impact on the Watchman device. We propose that when selecting the size of the LAmbre's outer disk, the operator should take into account the pre-ablation measurements or choose a larger outer disk if the only measurements were taken after ablation. In addition, the occlusion-first operation strategy in the combined procedure may reduce the risk of PDL because it minimizes the probability of improper LAA outer ostium measurement caused by edematous tissue at the ridge region (31). The relationship between PDL and thromboembolic events is controversial, with Holmes et al. (7) stating that there is no evidence that minimal PDL is associated with postoperative thromboembolic events while others suggest that incomplete occlusion increases the risk of thrombosis compared with complete occlusion (32). In our study, although the incidence of PDL was higher in the LAmbre-combined group than the Watchman-combined group, there were no thromboembolic events in the LAmbre-combined group, whereas two TIA and one DRT occurred in the Watchman-combined group were not detected PDL at TEE follow-up. To the best of our knowledge, none of these events were associated with the PDL.

Delayed PE is a serious complication, and a recent randomized clinical trial showed that delayed PE events are more likely to occur with “disk” devices than with “plug” devices (33). The rates of delayed PE were comparable between the Watchman-combined group and the LAmbre-combined group in our study. A possible explanation for this result is that we adopted a modified implantation method in the LAmbre-combined group: the umbrella was initially deployed half open outside the LAA and fully deployed to the landing zone, facilitating the full opening of the umbrella and thus reducing the mechanical force against the LAA wall (34).

In this retrospective study, the incidence of major adverse events during the follow-up period was not significantly different between the two groups (Figure 2) and similar to the results of previous studies [Watchman: 2.8 (4) and 4.1% (8); LAmbre: 2.6 (9) and 3.8% (8)]. The annual incidence of all-cause stroke and TIA was 1.0% in the Watchman-combined group and 0% in the LAmbre-combined group, which declined to 78% and 100% as the expected stroke rate at the same CHA_2_DS_2_-VASc score, respectively (Figure 1) (18). In this study, both groups had a low rate of bleeding events during follow-up, probably because more than 90% of patients in both groups (93.2% in the Watchman-combined group vs. 95.6% in the LAmbre-combined group) discontinued OAC treatment 6 months after the combined procedure. The rate of bleeding events was lower in both groups compared to previous studies [Watchman: 3.6% (35); LAmbre: 4.1% (8)], and the annual incidence of major bleeding decreased to 64% in the Watchman-combined group and 69% in the LAmbre-combined group, compared with the expected bleeding rate at the same HAS-BLED score (Figure 1) (19).

Previous studies have shown a heavier AF burden in the blanking period when LAAC was combined with PVI, but whether LAA occlusion had a significant effect on long-term ablation success was not determined (36). In the present study, AF recurrence rate was not statistically different at 6-month follow-up between the two groups. Additionally, the AF recurrence rate was not significantly higher in either group than in previous combined procedure studies [23.8 (1), 23 (12), 26.8% (31)].

### Limitations

This study had several limitations. First, the sample size of this study was small, limiting the generalization of the findings. Second, this was a retrospective study, and the patients were followed for a relatively short time; thus, long-term risk reduction could not be predicted. Third, the operators tended to choose the Watchman device in the early phase of this study, which resulted in longer follow-up in the Watchman-combined group than in the LAmbre-combined group. However, it did not affect the analysis since the adverse events rates were calculated as the number of events per 100 patient-years of follow-up. Fourth, some patients did not undergo TEE during the follow-up period because of poor tolerance (due to advanced age) or personal preference, which may have affected the duration of OAC. For most patients, only one TEE examination was performed during the follow-up period, and small DRT or PDL may have been missed; thus, the number of DRT and PDL reported may have been less than the actual number.

## Conclusion

The efficacy and safety of Watchman and LAmbre devices in the combined procedure were comparable. The rate of PDL was significantly higher at 3–6 months TEE follow-up than in the intraprocedural period, and it was higher in the LAmbre-combined group than the Watchman-combined group.

## Data availability statement

The original contributions presented in the study are included in the article/supplementary material, further inquiries can be directed to the corresponding author/s.

## Ethics statement

The studies involving human participants were reviewed and approved by Ethics Committee of the Second Affiliated Hospital of Wenzhou Medical University. The patients/participants provided their written informed consent to participate in this study. Written informed consent was obtained from the individual(s) for the publication of any potentially identifiable images or data included in this article.

## Author contributions

J-YK, Y-NL, and Y-CL contributed to the conception and design of the study. L-SJ, J-YF, LL, Y-LC, and Y-XQ organized the database. JX and W-KL performed the statistical analysis. J-YK wrote the first draft of the manuscript. J-YK, L-SJ, and Y-NL wrote sections of the manuscript. All authors contributed to the manuscript revision, read, and approved the submitted version.

## Conflict of interest

The authors declare that the research was conducted in the absence of any commercial or financial relationships that could be construed as a potential conflict of interest.

## Publisher's note

All claims expressed in this article are solely those of the authors and do not necessarily represent those of their affiliated organizations, or those of the publisher, the editors and the reviewers. Any product that may be evaluated in this article, or claim that may be made by its manufacturer, is not guaranteed or endorsed by the publisher.

## Abbreviations

RFCA: Radiofrequency catheter ablation
LAA: Left atrial appendage
LAAC: Left atrial appendage closure
NVAF: Non-valvular atrial fibrillation
AF: Atrial fibrillation
OAC: Oral anticoagulant
TEE: Transesophageal echocardiography
PVI: Pulmonary vein isolation
PE: Pericardial effusion
DRT: Device-related thrombus
SAT: Single antiplatelet therapy
PDL: Peri-device leakage
TIA: Transient ischemic attack
LAR: Left atrial ridge.
